# Progress in Surgery

**Published:** 1898-03-26

**Authors:** 


### Progress in Surgery.

### SURGERY OF THE SPLEEN AND PANCREAS.

**Cavernous Angioma** of the spleen is very rare. Homans[1] reports a case in a woman æt. 22. She had been tapped many times for ascites, but the fluid was always bloody. When Homans first opened the peritoneal cavity seventeen pounds of bloody ascitic fluid were removed. Between the hepatic and splenic flexures of the colon and surrounded by omentum was a tumour which appeared irremovable, but was ultimately taken away with a supernumerary spleen. The patient recovered, but four months after another operation was performed because the fluid had reaccumulated. This time the spleen, which was enlarged, was removed. The patient died of shock 20 hours after operation. The structure both in the spleen and in the peritoneum seemed to be of the same character, viz., a cavernous angioma.

**Wandering Spleen** exhibits, says Sutton,[2] more mobility than a movable kidney. It is most common in women, and generally in those that have borne children. Its dangers are: (1) Rupture of the stomach or duodenum. (2) Intestinal obstruction. (3) Abscess of spleen. (4) Rupture of spleen. It may be confounded with ovarian cysts, renal tumours, malignant tumours of the omentum, movable kidney, and uterine myoma. Splenectomy for this condition is regarded as justifiable, because the rate of recovery is equal to that of ovariotomy in skilled hands, and no remote consequences have been reported. Greiffenhagen[3] reports a case of splenopexy for this condition.

**Splenectomy** has been performed by Jonnesco[4] six times for enlargement in association with malarial fever, and once for a very large suppurating hydatid cyst. In all but one, recovery was complete and speedy. In cases of malaria the operation is indicated in the absence of leukæmia, of extensive adhesions of the organ, and of well-marked cachexia, whenever medical treatment has failed, and need not be influenced by the size of the organ. For some days after operation there is an enormous relative increase of white corpuscles, but subsequently the proportion between white and red corpuscles becomes normal.

**Pancreatic Cysts**, says Penrose,[5] can rarely be excised, and the usual treatment is by incision and drainage. Zweifel states that extirpation has been performed 12 times with five deaths; and of 31 cases in which incision and drainage have been done only two died, but 10 of these suffered from a more or less permanent fistula. Though ordinarily sterile, the fluid of these cysts is not uniformly so, and therefore it is best to refrain from aspiration lest any fluid not sterile should escape and set up a fatal peritonitis. The fluid found in a case reported by the author rapidly transformed starch into sugar, and emulsified neutral fat. Its action on proteids was uncertain.

**Pancreatitis**.—In three cases recorded by Simon and Stanley,[6] bacteriological examination of the tissues of the pancreas and the fluid contained in the surrounding cellular tissue showed the presence of different micro-organisms. One of the most distinctive was a somewhat slender bacillus, with rounded ends which did not stain by Gram's method. Bacterium coli commune was also obtained by culture. Taking into consideration the manner in which the stomach and duodenum showed intense inflammation, with involvement of the head of the pancreas—*i.e.*, the adjacent portion—the tail practically escaping, the question may be raised as to whether we may not have to deal with an infective gastro-duodenitis, extending into the surrounding tissues. Fitz divides these cases into: (1) Pancreatic hæmorrhage; (2) hæmorrhagic pancreatitis; (3) acute pancreatitis, which might stop short of or reach a gangrenous condition. The symptoms are sudden and severe pain in the upper part of the abdomen, shock which may kill the patient with the rapidity of cerebral hæmorrhage, and vomiting which continues unrelieved until stopped by death. Most cases are treated by laparotomy, because of the difficulty of making a diagnosis; but when once the disease has started no treatment is effectual. In the earlier stages it is possible that by vigorously treating gastro-duodenal catarrh by salol and other disinfectants we may diminish the likelihood of pancreatic infection. McPhedran[7] refers to a case of pancreatitis followed by the formation of a cyst. The patient recovered after operation.

[1] Annals of Surg., June, 1897, p. 732. [2] Brit. Med. Jour., 1897, vol. I.; and Amer. Med. Surg. Bullet., April 25th. 1897. [3] Amer. Journ. Med. Sci., April, 1897, p. 485. [4] Progrès Med., No. 12, 1897; and Brit. Med. Journ., April 24th, 1897. [5] Annals of Surg., April, 1897, p. 491. [6] Lancet, May 15th, 1897, p. 1325. [7] Med. Rec., June 5th, 1897, p. 822.

